# How storage post sampling influences the stability of sebum when used for mass spectrometry metabolomics analysis?

**DOI:** 10.1038/s41598-024-71598-7

**Published:** 2024-09-17

**Authors:** Caitlin Walton-Doyle, Eleanor Sinclair, Humayra Begum, Katherine A. Hollywood, Drupad K. Trivedi, Perdita Barran

**Affiliations:** The Michael Barber Centre for Collaborative Mass Spectrometry, The Department of Chemistry The Manchester Institute for Biotechnology, The University of Manchester, Manchester, M1 7DN UK

**Keywords:** Analytical biochemistry, High-throughput screening, Mass spectrometry, Metabolomics, Biomarkers

## Abstract

Sebum is a biofluid excreted by sebaceous glands in the skin. In recent years sebum has been shown to contain endogenous metabolites diagnostic of disease, with remarkable results for Parkinson’s Disease. Given that sebum sampling is facile and non-invasive, its potential for use in clinical biochemistry diagnostic assays should be explored including the parameters for standard operating procedures around collection, transport, and storage. To this aim we have here investigated the reproducibility of mass spectrometry data from sebum in relation to both storage temperature and length of storage. Sebum samples were collected from volunteers and stored for up to four weeks at a range of temperatures: ambient (*circa* 17 °C), −20 °C and −80 °C. Established extraction protocols were employed and samples were analysed by two chromatographic mass spectrometry techniques and data investigated using PCA, PLS-DA and ANOVA. We cannot discriminate samples as a function of storage temperature or time stored in unsupervised analysis using data acquired via TD–GC–MS and LC–IM–MS, although the sampling of volatiles was susceptible to batch effects. This study indicates that the requirements for storage and transport of sebum samples that may be used in clinical assays are less stringent than for liquid samples and indicate that sebum is suitable for remote and at home sampling prior to analysis.

## Introduction

We have recently shown that sebum is a readily accessible biofluid which contains endogenous metabolites that vary in abundance due to the health or disease status of the individual sampled^1–4^. In the course of our exploration of sebum as a diagnostic biofluid we have commonly sampled remote from the analytical laboratories. This means that the samples spend differing times getting to the test laboratory. The primary objective of this study was to explore the variability of features found using both TD–GC–MS and LC–MS after storage at different temperatures and for differing times. Our work to date, and the general observation that odour remains trapped in sebum, has allowed us to hypothesize that diagnostic features in sebum are more robust to storage conditions than other biofluids. Here we systematically investigate the effect of storage time and temperature on sebum when analysed using GC–MS and LC–MS discovery workflows. In this controlled study, we sought to provide evidence regarding the effects of sebum sample storage on its use in mass spectrometry analysis using sebum from healthy volunteers.

Biofluids are used in healthcare settings to diagnose disease, delineate progression and monitor response to treatment. In earlier times, observable changes in the appearance and odour of biofluids were the primary diagnostic approach for physicians^5,6^. Nowadays, modern medicine has strict and established operating procedures^7^. Blood, a widely used diagnostic biofluid has rigorous standardised protocols for collection, separation into the two major forms: serum and plasma, and for subsequent storage (recommended at −80 °C or in cryogenic vaults at prior to analysis)^8^. Biobanks or local clinical resources used for such storage have high associated energy costs of maintaining the low temperatures to preserve the samples integrity^9^. These high running costs curtail the availability of biobanks to low and middle income (LMIC) countries, which in turn results in fewer discovery and validation biomarker studies from cohorts from lower economically developed countries^10^. Successful biofluid sampling that does not require cold storage has been developed for some clinical diagnostic methods. In several cases these are used for population wide screening, for example the infant heel prick assay for metabolic disorders where dried blood spots can be screened by mass spectrometry^11^. Another example is at home faecal sampling for bowel cancer which is applied in the UK to everyone aged 60 or over and screens for blood in stools^12^. We have recently developed mass spectrometry-based metabolomic methods to discover biomarkers from sebum and here we explore how this biofluid may be suited to similar population wide screening.

Sebum is a oily substance secreted by the sebaceous glands in the dermis and located all over the body, with the exception of palms of hands and soles of feet^13^. It has many roles, including providing an antimicrobial film, protecting against dehydration, thermoregulation, photoprotection and transporting antioxidants to the skin's surface^14–16^. The matrix of sebum contains many lipid classes: glycerides (30–50%), free fatty acids (FFA, 15–30%), wax esters (WE, 26–30%), squalene (12–20%), cholesterol esters (CE, 3–6%) and cholesterol (1.5–2.5%)^17^. The sebaceous gland is supplied with blood and is connected to the lymphatic system^18^. As such, excreted sebum will also contain endogenous compounds, which given its composition are likely to be enriched in lipophilic components. Our secretion of sebum alters throughout our lifespan. Newborns are born with excessive sebum often observable on their scalp. Sebum production then reduces until adolescence, when it is again elevated^19^. After this, there are no significant changes as we age, except for a decrease in secretion in women after menopause^20^. Changes in sebum production and composition have primarily been investigated to better understand skin conditions such as acne vulgaris and rosacea. Excessive sebum production (seborrhoea) is a hallmark of Parkinson’s Disease (PD) and is used as a support for diagnosis by clinicians^21,22^. This increased production of sebum in people suffering from PD was first reported almost 100 years ago^23^. Recently we have shown that changes in the lipid composition of sebum^3^ as well as in the abundance of volatile compounds are hallmarks of PD^1,2^, which can be used diagnostically and prognostically. Analysis of sebum also provides insights to important metabolic processes in disease progression which is not just applicable to PD. We have observed lipid dysregulation in people infected with SARS-CoV-2 using sebum samples, where COVID-19 positive patients showed depressed lipid levels^24^. These promising foundational studies show that sebum can be used both to provide a better understanding of disease mechanisms and identify diagnostic targets.

One of the most attractive features of sebum as a potential diagnostic biofluid is that the sampling is non-invasive. This said, compared with blood, urine and saliva, sampling and storage conditions suitable for sebum prior to a clinical biochemistry analysis—for example by Mass Spectrometry—have not yet been thoroughly investigated. In terms of collection material, sebum can be sampled onto different surfaces including gauze^1–3^, glass beads^25^ and sebutape^TM^^26^, and compared to the ubiquitous test tubes used for blood, urine and saliva, such collection systems lack standardisation, and different materials display different background signatures^27^.

Sebum sampling has potential to be performed at home as well as in clinical environments, the former presenting a lower cost option for population screening, as with the fecal and blood spot examples provided above^11,12^. In our previous studies, samples have been collected in clinic or self-collected at home, placed in sealed Ziplok™ bags and transported to research facilities using domestic mail rather than specialist courier. Once arriving in the research lab, samples have been placed in cold storage (−80 °C) for up to 5 years prior to extraction and analysis. The time between sampling and this cold storage is usually 2–5 days but has been longer in some cases. While, our data has shown distinct biological effect, underlying shifts in metabolome or lipidome as a result of storage conditions remain to be investigated. The fact that none of our studies to date have shown any bias between samples collected and cold stored immediately versus those that spent more time in ambient conditions, as well as no difference based on the amount of time in cold storage led us to investigate the effects of storage on sample integrity.

Volatile metabolites may require different treatment than more researched biofluids and may escape from samples in some environments. To counter this, it is a well observed anecdote that unwashed baby clothes continue to smell of babies many years after they have been worn. This observation suggests that sebum acts as a preservative for volatile components. To further the use of sebum as a diagnostic biofluid, it is desirable to determine the criteria for collection media, transportation and storage prior to analysis that will ensure the integrity of the sample.

To provide evidence for the above hypothesis, we examined sebum sampled cotton swabs and gauze samples stored at different temperatures over defined time points to better understand possible degradation patterns to direct future sebum storage condition standardisation. We analysed volatile compounds by headspace sampling in combination with Thermal Desorption–Gas Chromatography–Mass Spectrometry (TD–GC–MS) and less volatile components following solvent extraction with Liquid Chromatography–Ion Mobility–Mass Spectrometry (LC–IM–MS).

## Methods

### Participants and samples

**Fig. 1:**
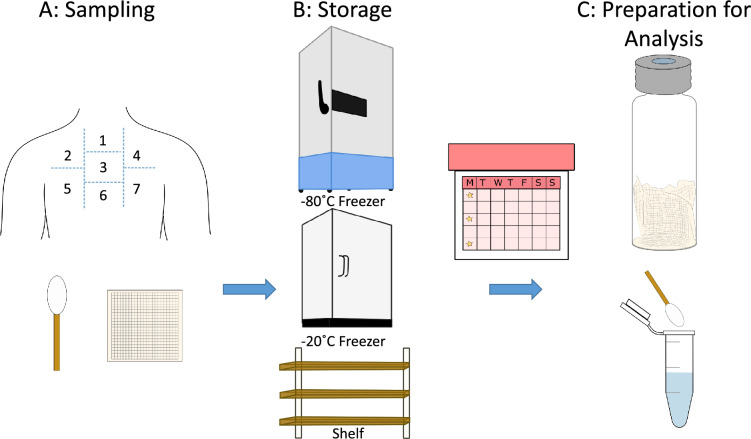
Each participant was sampled on seven sites on the upper back using both gauze and cotton swabs (**A**). All samples were transported to the lab and placed in different temperature storage environments namely a −80 °C freezer, a −20 °C freezer and a shelf in the laboratory (**B**). Samples were processed following storage at different time points: zero weeks (t_0_), two weeks (t_2_) and four weeks (t_4_). The gauze was analysed by headspace analysis, whereas the cotton swabs were extracted and batched for LC–IM–MS analysis (**C**).

Sebum samples were taken from the upper back of participants (*n* = 8, Table 1) following established methods which we describe here briefly^1–3^. Individuals were asked not to shower for at least 24 h prior to collection. Gauze and cotton swabs were rubbed across distinct areas of the skin on the upper back to collect sebum (Fig. 1A). Samples were collected at home from individuals who each provided 14 samples, using gauze (*n* = 7) and cotton swabs (*n* = 7) taken by themselves or another individual. Sampled gauze swabs were sealed in individual Ziplok™ bags immediately after collection whilst cotton swabs were stored in their original individual plastic tube holder. The gauze and cotton swab samples were taken on different days to ensure there was sufficient sebum on skin. Once all samples were collected, they were transported to our research labs and placed in a box, meaning collected samples were at ambient temperatures for up to 48 h prior to the start of the experimental period. Additionally, ethnicity was recorded for each participant which included; Bangladeshi (*n* = 4), Caucasian (*n* = 3) and Afro Caribbean and Caucasian (*n* = 1). None of the participants were smokers and did not consume alcohol 24 h prior to sampling. Ethical approval for this project (IRAS project ID 191917) was obtained by the NHS Health Research Authority (REC reference: 15/SW/0354). Informed consent was received from all participants prior to their enrolment in the study. All experiments were performed in accordance with relevant guidelines and regulations as set out by the UK NHS Health Research Committee.

**Table 1:** Summary of participant information.

Metadata (*n* = 8)	
Gender (Female:Male)	1:1
Age (years)	33.6 ± 14.5*
BMI	22.0 ± 1.8*

*Data reported as mean ± standard deviation.

### Experimental design

Materials used are listed in the supplementary information. Gauze swabs were analysed directly by TD–GC–MS and cotton swabs were solvent extracted and analysed by LC–IM–MS, sample preparation and analytical methods are detailed the supplementary information including how we are able to obtain a substantial number of comparable significant features from every sample regardless of variations in sample collection from individual to individual. The experimental design is shown in Fig. 1, with the storage conditions listed in Table 2.

**Table 2:** Designation of samples to experimental conditions and analytical techniques.

Storage temperature	Time point (weeks)	Analytical techniques
Ambient (c.17 °C)	0, 2, 4	LC–IM–MS, TD–GC–MS
− 20 °C	2, 4	LC–IM–MS, TD–GC–MS
− 80 °C	2, 4	LC–IM–MS, TD–GC–MS

A gauze swab and a cotton swab from each participant was analysed at each temperature and time point to permit analysis with both MS methods. For LC–IM–MS analysis of sebum on cotton swabs, the samples were extracted at t_0_, t_2_ and t_4_. Dried extracts were stored at −80 °C until analysis and subsequently all samples were reconstituted and analysed together. Dynamic headspace sampling to capture and concentrate volatile compounds for TD–GC–MS analysis is performed directly from the gauze swab, so these samples were analysed at each time point in three batches (t_0_, t_2_ and t_4_) separated by two-week intervals.

### Data analysis

Data pre-processing and feature filtering steps are detailed in SI 3. For the resultant features, MetaboAnalyst^28^ was utilised for statistical analysis, where all data was log transformed and autoscaled. The primary approaches used to analyse data were Principal Component Analysis (PCA) and Partial Least Squares-Discriminant Analysis (PLS-DA). Significant features were found using Analysis of Variance (ANOVA), with FDR and adjusted *p*-values ≤ 0.05. The samples grouped by participant, and investigation into the number of features and signal intensity across conditions are located in SI 4 and 5, and Figure SI 2 and 3.

## Results and discussion

### Investigation of sebum stability with respect to storage temperature

Samples were first categorised by the temperature at which they were stored, defined as four categories; fresh sebum samples which were not stored (t_0_), ambient laboratory temperature (c. 17 °C), −20 °C and −80 °C. The length of time in storage was not considered here for any samples.

**Fig. 2:**
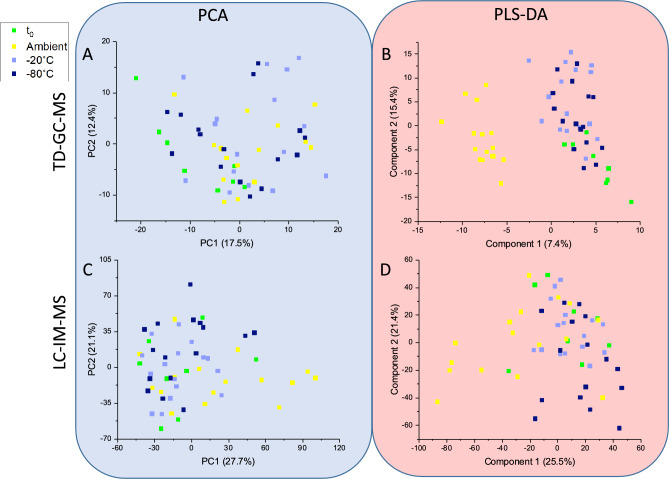
PCA (**A** & **C**) and PLS-DA (**B** & **D**) scores plots of all samples separated according to storage temperature for both TD–GC–MS (**A** & **B**) and LC–IM–MS (**C** & **D**) analyses. Initial samples are represented in green, ambient samples in yellow, −20 °C samples in light blue and −80 °C samples in dark blue.

TD–GC–MS: Unsurprisingly, unsupervised PCA scores plot (Fig. 2A) show no separation between the groups. Supervised classification using PLS-DA was then performed (Fig. 2B), which again did not show clear separation between the four groups, however, some clustering can be noted of the samples stored at ambient temperature against the combined t_0_ fresh sebum, −20 °C and −80 °C samples. The PLS-DA could not be validated by cross validation or permutation testing (Figure SI 4), indicating the model was overfitted and the small amount of clustering for samples at ambient temperature is an effect of this, rather than any real effect.

Of the 471 robust features in the TD–GC–MS data matrix 89% (417) were found not to be significant (*p* > 0.05) with relation to storage temperature by ANOVA. A histogram was plotted to investigate spread of the significant features by chromatographic retention time (RT) (Figure SI 5) and no trend was observed.

The 54 significant features (*p* ≤ 0.05) were investigated and putatively identified as alkanes (*n* = 11) and fatty acids (*n* = 7) the remaining features could not be identified. Of these, 25 showed consistent regulation across temperatures, 19 were downregulated from ambient to −20 °C and from −20 °C to −80 °C and 6 upregulated.

LC–IM–MS: No clustering was seen for samples based on storage temperature, particularly in the unsupervised PCA (Fig. 2C). PLS-DA scores plot (Fig. 2D) displays broad overlap between the four groups, with some grouping of the ambient samples. The PLS-DA could not be validated—the low accuracy, R^2^ and Q^2^ indicate that the PLS-DA is overfitted (Figure SI 6).

**Fig. 3:**
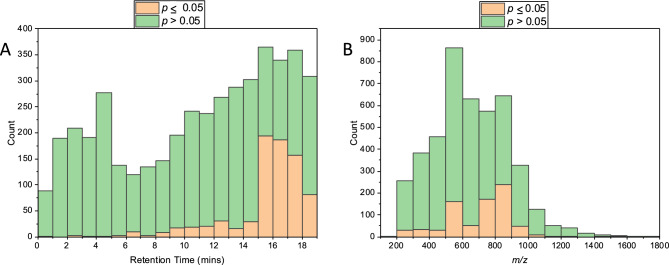
Histograms showing the LC–IM–MS significant features (*p* ≤ 0.05) as a function of storage temperature. Figure (**A**) shows the features distribution with respect to retention time, and (**B**) with respect to m/z values.

Despite the inability to separate any sample set, 779 of the 4399 total features (~ 18%) are significantly different in expression (*p*-value ≤ 0.05). This distribution of non-significant *vs.* significant features was investigated in terms of both RT (Fig. 3A) and *m*/*z* (Fig. 3B) to infer any chemical properties of these analytes.

Approximately 80% of the significant features elute between 15 and 19 min and are found across the *m/z* range 500–900 (Fig. 3). Figure 3A demonstrates that for early retention times we note only a very small proportion of significant features, this rises for later retention times (Fig. 3A). Between 15–18 min of the LC gradient, the mobile phase composition (approximately 80% IPA:ACN:H_2_O) favours elution of hydrophobic lipid-like species. This suggests that lipid-like molecules are more likely to vary both in presence and amount as a function of temperature of storage than hydrophilic features that elute earlier.

Of the top 20 significant features, 14 were seen to increase at colder temperature, two decreased and four showed no trend. A list of these features is reported in the Table SI 1, 14 of the 20 are in the RT range 15–19 min, which incidentally are the 14 that increase at −80 °C.

### Investigation of sebum stability with respect to storage time

Next, samples were grouped according to storage time: initial (t_0_), two weeks (t_2_) and four weeks (t_4_), irrespective of storage temperature. It should be noted, the PLS-DA scores plots in the following section were inputted with class order ranked (maximum variance was calculated with the time points in order).

**Fig. 4:**
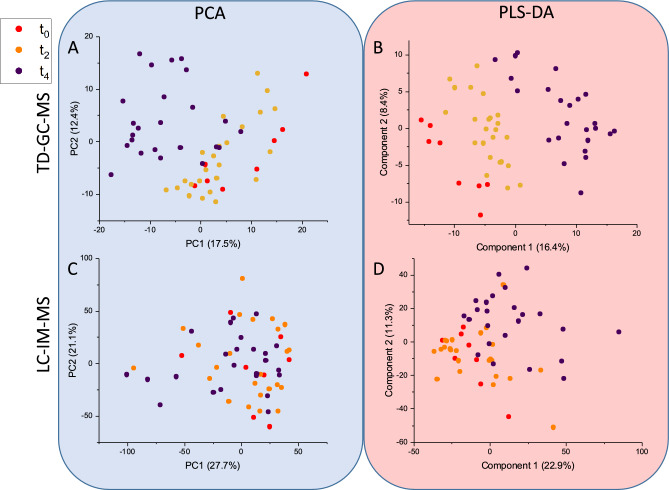
PCA (**A** & **C**) and PLS-DA (**B** & **D**) scores plots of the samples separated according to time post sampling for both TD–GC–MS (**A** & **B**) and LC–IM–MS (**C** & **D**) analyses. Initial values (t_0_) are represented in red, t_2_ in orange and t_4_ in purple.

TD–GC–MS: As the samples were ran in three separate batches at the three time points, they are subsequently referred to as batches for the GC data. PCA (Fig. 4A) and PLS-DA (Fig. 4B) scores plots were generated to examine any measured differences in the TD–GC–MS data based on length of time in storage. The PCA displayed slight clustering with overlap between batches and the PLS-DA indicated the batches almost completely separated, this model could be validated (Figure SI 7). Of the 471 features in the data matrix, 175 were found to be significant (*p* ≤ 0.05) by ANOVA. The significant features when comparing time points were investigated further and the top 20 are reported in Table SI 2. Of the significant features, 36 were found to increase over the four-week period at each storage time interval, 89 decrease, and 50 show no consistent pattern. Putative identifications were assigned to the features, among which 35 were identified as FAMEs and 37 as alkanes.

LC–IM–MS: PCA (Fig. 4C) and PLS-DA (Fig. 4D) were executed with samples separated by time point and the resultant scores plots are displayed. The PCA plot presents no separation between groups. In PLS-DA, intra-group variation was found to be the least within t_0_ samples and the highest in t_4_ samples, again there was no separation between time points, the validation and permutation of the PLS-DA is displayed in Figure SI 8 which indicate the model is overfitted.

ANOVA found only 177 features to be significant when *p* ≤ 0.05 which is just 4% of the total features and an increased *p*-value threshold at 0.1 still only accounts for 5% of features. 50% of the significant features (*p* < 0.05) are detected in the *m/z* 800–1000 range, a small fraction of the total number of features in this mass range. Significant features (*p* < 0.05) were investigated, the 20 with the lowest *p*-values are listed in Table SI 3. Of the 177 features, 26 were seen to increase over the four-week period, 14 decreased and 137 showed no consistent pattern over the three time points.

For TD–GC–MS, the samples were analysed in batches separated by two weeks, unlike the LC–IM–MS samples which were extracted at intervals, with extracts stored and randomised, reconstituted and analysed together. Due to this we investigated instrument stability over the three batches. The sample and SST data were deconvoluted in separate analyses and a PLS-DA was executed to compare SST to sample data shown in Figure SI 9. The samples and the SST injections both separate by batch, which suggests that any differences we see in the TD–GC–MS data over the four weeks is due to instrument shift and batch variation rather than composition of volatile signature.

### Significant features by participant

The individual participant effects were examined for the most significant features for each model and are detailed in section SI 6 and Figure SI 10–13. This displays that trends across the participants are in agreement with the observations in the above analysis, indicating that although the sample set was relatively small, the results from individuals are consistent with the overall results shown.

## Conclusions

The data above indicates that we cannot distinguish samples on the basis of the temperature under which they have been stored from a mass spectral profile. For gauze, we note that most of the features are not affected by storage temperature, and there is no trend to class of significant features which indicates it is not due to bioprocesses.

When we compare LC–MS data with regards to storage temperature here to that found in experiments that are discriminating between a disease and control group^19^ 38% of the features found in that analysis were significant with *p* ≤ 0.05, compared to only 18% here. The few distinguishing features here are mostly highly similar chemical class (retention time), in contrast to the LC–IM–MS Parkinson’s Disease diagnostic work where we see significant features do not cluster with RT (on a slightly altered LC gradient between 20 and 29% of the significant features are found in each quarter of the LC run)^3^. This shows that in our use of sebum as a diagnostic biofluid we do not see the same patterns in significant features as we do here with respect to storage temperature and thus indicates that storage temperature would not affect the diagnostic capabilities of sebum.

For storage time, in LC–IM–MS analysis there is less discrimination based on length of storage and a much smaller proportion of features found to be significant than for the TD–GC–MS analysis. As we know the large variation in TD–GC–MS data is due to batch-to-batch variation, it is expected this would not be seen in LC data where samples were ran continuously over two days. The very small proportion of features found to be significant, with no commonality between classes or regulation, and the lack of any separation in scores plots indicates that over time, extracted sebum shows good stability.

For TD–GC–MS data, any variation over the four-week period is attributed to instrument drift. This underlines the importance of continuous analysis in metabolomics experiments, as well as the use of robust SSTs.

We conclude that the temperature at which a sample is stored and the time that the sample is stored for, will not significantly alter results when using sebum as a diagnostic biofluid. This is in support of our prior analysis of sebum samples from over 650 participants, where we saw no correlation based on sampling site nor time from sampling to analysis; *n* = 190 (GC–MS)^1,2^ *n* = 341(LC–MS)^3,24^ and *n* = 150 (PSI (paper spray ionisation) MS)^4^. This indicates it has potential use for sampling remote from analysis, for example at home, with the samples then posted to the test laboratory.

## Supplementary Information

Supplementary Information.

## Data Availability

Raw data sets generated during the current study are available from MetaboLights Repository https://www.ebi.ac.uk/metabolights/search with the study Identifier MTBLS7946.
