# Supplementary material for: How storage post sampling influences the stability of sebum when used for mass spectrometry metabolomics analysis?
Source: Sci Rep. 2024 Sep 17;14:21707. doi: 10.1038/s41598-024-71598-7 (PMC11408688; doi:10.1038/s41598-024-71598-7)
Supplement: Supplementary file 1 — Supplementary Information. [file 41598_2024_71598_MOESM1_ESM.docx]

Supplementary Information in support of

How Storage Post Sampling Influences the Stability of Sebum when used for Mass Spectrometry Metabolomics Analysis

Caitlin Walton-Doyle, Eleanor Sinclair, Humayra Begum, Katherine A. Hollywood, Drupad K. Trivedi and Perdita Barran*

The Michael Barber Centre for Collaborative Mass Spectrometry, The Manchester Institute for Biotechnology, Department of Chemistry, The University of Manchester, M1 7DN

Corresponding author: Perdita.barran@manchester.ac.uk

SI 1: Materials

Sebum samples were collected using gauze swabs (Arco, UK) placed in sample bags (GC Healthcare Whatman, UK) and Sterilin Q-Tip swabs (VWR International, USA). Chemicals and materials used in the study were: microcentrifuge tubes 2mL (Eppendorf, UK), methanol, acetonitrile, formic acid, 2- propanol, ammonium formate (Fisher Scientific), HiPerSolv Chromanorm absolute ethanol (VWR International, USA), Chromasolv LC-MS grade water (Honeywell, USA), 20mL headspace vials (Gerstel, Germany), TENAX TA thermal desorption tubes and TENAX TA for CIS 4/6 liner for TDU (Gerstel, Germany). The SST for TD-GC-MS analysis was a mixture of seven compounds from Sigma-Aldrich (L- carvone, δ-decalactone, ethyl butyrate, ethyl hexanoate, hexadecane, nonane and vanillin) in MeOH:EtOH (9:1).

SI 2: Sample Preparation and Analytical Methods

TD-GC-MS: Gauze swabs were transferred from sample bags to 20 mL headspace vials and were analysed by Dynamic Headspace (DHS) TD-GC-MS. Samples were randomised and blinded in each batch and run on an Agilent 7890A GC paired with an Agilent 5975 MSD, operated with a Gerstel MPS dual head system.

Samples were incubated in the DHS (80 ˚C, 10 min) to preconcentrate volatile analytes in the headspace. Following this, dry nitrogen (1 L, 70 mL min^-1^) was purged through the headspace to collect analytes onto a Tenax adsorbant tube (40 ˚C). This tube was then transported to the Thermal Desorption Unit (TDU) located in the GC inlet. The TDU temperature gradient was: 30 ˚C for 1 minute, heated to 280 ˚C at a rate of 720 ˚C·min^-1^ where it was held for 5 minutes. The analytes desorbed from the TDU into the cooled injection system (CIS). The CIS temperature gradient was: 10 ˚C for 0.5 minutes, heated to 280 ˚C at a rate of 720 ˚C·min^-1^. It was held at 280 ˚C for 5 minutes.

The GC separation was performed using an Agilent VF-5MS column (30m x 0.25mm x 0.25um) with a flow rate of 1mL·min^-1^. The oven gradient was: 40 °C held for 1 min, 25°C·min^–1^ to 180°C, 8 °C·min^–1^ to 240°C where it was held for 1 min, 20 °C·min^–1^ to 300°C held for 2.9 min. The total run time for each sample was 21 minutes. The transfer line was kept at 300˚C, the EI source at 230 ˚C and the quadrupole at 150 ˚C. The MSD mass range scanned was *m/z* 30-800.

As each participant gauze is analysed directly there is no simple way to pool a biological QC. A mixture of scented compounds was used as a system suitability test (SST) to account for instrument drift and analytical reproducibility over time. These SST samples (20 µL) were injected at the start and then after every 5^th^ injection as well as at the end of each batch.

LC-IM-MS: The wooden stems of the Q-tip swabs were snapped so they would fit in 2mL microcentrifuge tubes. Methanol (1 mL) was added to each followed by vortex mixing (10 sec), sonication (5 min) and a second vortex mixing (10 sec). Each Q-Tip was removed from it’s tube leaving the metabolite rich methanol extract. Samples were centrifuged (15 min, 13,800 *g*) and 600 µL was transferred to a new tube. 100 µL of the remaining liquid was used to create a biological pooled QC sample. Extracts were vacuum centrifuged for 4 hr at ambient temperature, and the dried fraction stored at -80 ˚C until analysis. A blank Q-Tip were extracted following the same protocol as a sample at each time interval. After all samples were collected and extracted, 200 µL methanol was added to extracts, pooled QC and blank samples. They were then vortex mixed (10 sec), sonicated (5 min) and centrifuged (15 min at 13, 800 *g*). The supernatant (160 µL) was submitted for LC-IM-MS analysis.

LC-IM-MS analysis was performed on an Acquity UPLC I-Class coupled to a Cyclic IMS Mass Spectrometer (Waters Corporation) fitted with an electrospray ionisation (ESI) source. Data were acquired using MassLynx 4.2. An ACQUITY UPLC CSH C18 column (1.7 µm, 2.1x100 mm) with an ACQUITY CSH C18 1.7 µM VanGuard pre-column were used for separation. The mobile phases were: A: acetonitrile:water (*v/v*, 60:40) and B: isopropanol:acetonitrile:water (*v/v*, 85:10:5) both with 10 mM ammonium formate and 0.1% formic acid. The flow rate was set to 0.4 mL·min^–1^ and the gradient began at 90% A, it decreased to 50% A over 4 minutes, then to 30% A at 11.6 mins, 26% A at 11.7 mins, 13% A at 16 mins and 5% A at 18 mins. The composition then went to 90% A at 18.1 mins where it equilibrated until 24 minutes (total run time).

Full MS spectra were obtained across the mass range *m/z* 50-2000 and Leucine-Enkephalin (*m/z* 556.2766) was infused as a mass calibrant via the LockSpray setup. MS settings were: capillary voltage 2.0 kV, sampling cone voltage 40 V, source temperature 120 ˚C, desolvation temperature 550 ˚C and desolvation gas flow 1200 L/h. MS^e^ was used to collisionaly activate all analytes without *m/z* selection with a potential ramp from 15-45 V. ^1^

Pooled QC samples were used to check reproducibility of the instrument across batches. These ran at the beginning of each batch, every 5^th^ injection and at the end of each batch. 56 Participant samples across all temperatures and time points were randomised and split into two batches of 38 and 18 which were reconstituted on day of analysis. Additionally, the blank Q-Tip extracts were analysed in each sample batch for a reference of background, they were seen to cluster together and completely separate from samples and QCs in both PCA and PLS-DA models.

SI 3: Data Pre-Processing and Deconvolution

TD-GC-MS: SST data were used to gauge instrument replicability throughout and between each time point analysis. TICs were manually overlaid and no significant retention time shift or intensity change was observed. All data from participant samples were converted to open source mzXML format using ProteoWizard. Data were deconvolved in R using in house scripts and eRah packaging. Putative identifications (MSI level 2) were given by matching fragments with the GOLM database where any assignment with match factor <75 or as a TMS derivative was not considered identified. The output matrix had 471 features and their corresponding peak area for each sample. A multivariate approach was used to investigate differential volatile signatures.

LC-IM-MS: LC-IM-MS data were aligned and deconvolved in Progenesis QI metabolomics (Waters, Wilmslow). Retention times (RTs) were aligned against the most suitable QC. Peak picking was performed with a minimum peak width of 0.2 minutes which returned 17,731 compound ions. Any ions that were most abundant in blank samples were removed, as were any features that had a fold change < 1.2 between mean intensity of samples and blanks. Coefficient of Variance (CV) was calculated for ions in the QC injections, and any feature with a CV > 20 % were removed. 4,399 features remained in the data matrix which were used for statistical modelling.

Putative annotations (MSI level 2) were assigned using the Lipid Maps database (LMSD) and Lipid Blast database using a threshold window of 10 ppm for both the precursor and fragment ions. For investigation into the effect of time and temperature the QCs were included initially, once it was shown they clustered together and demonstrated the analytical reproducibility desired they were removed from analysis.

SI 4: Features by Participant

To ensure the variation we measure was not due to different volatile signatures on individuals the data was grouped by participant. None of the participants separated entirely from the others, though some clustering was observed in the GC data. Three participants (102, 201 and 202) were seen to separate together more from the others, they differed in ethnicity, sex and BMI and did not share any medical conditions. Though they were similar in age, other people the age bracket did not separate with them. The PLS-DA of both data sets is shown in Figure SI 1.


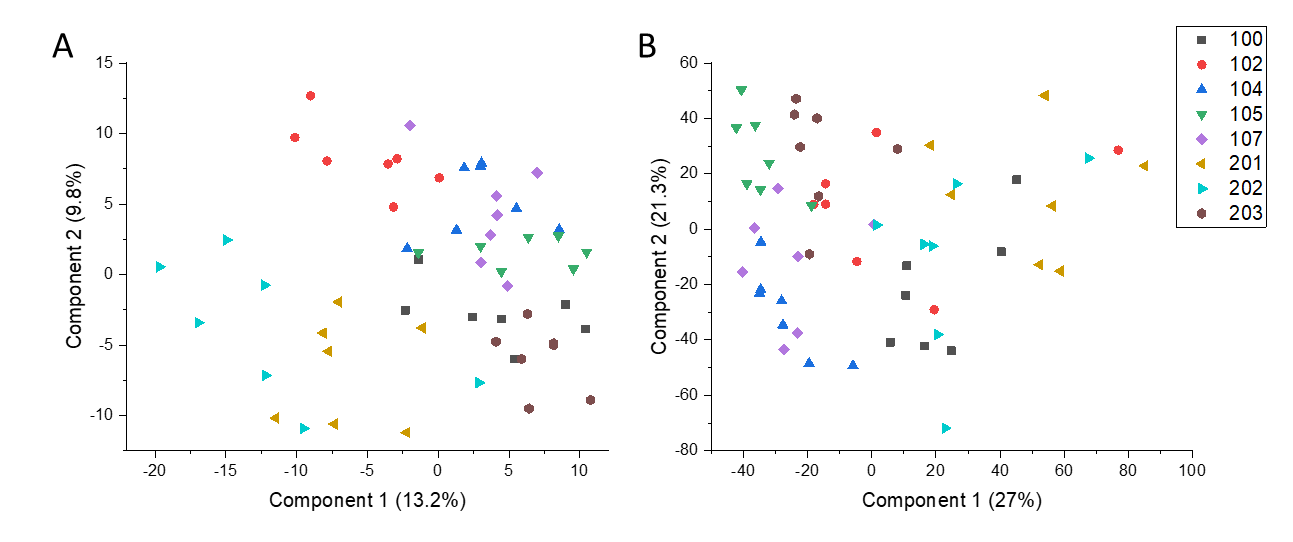


Figure SI 1: PLS-DA plots of TD-GC-MS data (A) and LC-IM-MS data (B) of the samples separated by participant.

SI 5: Sum of Peaks and Number of Features

Intensities of all features in the Total Ion Chromatogram (TIC) were summed for both TD-GC-MS and LC-IM-MS. In both analytical techniques there was no correlation between total TIC over time or storage temperature across the participants (Figure SI 2 and Figure SI 3 A). This also indicates that any variation in sampling methodology for example, sampling slightly different regions of the mid back and/or with different pressure or any variation of total sebum present on the back of each participant is also not significant as we have previously demonstrated.^2^ The number of features in LC-IM-MS analyses were also mapped (Figure SI 3 B) and it is displayed that 4 participants have a loss of features at 4 weeks at ambient temperature, in the most extreme case this is a still only a loss of 6%.


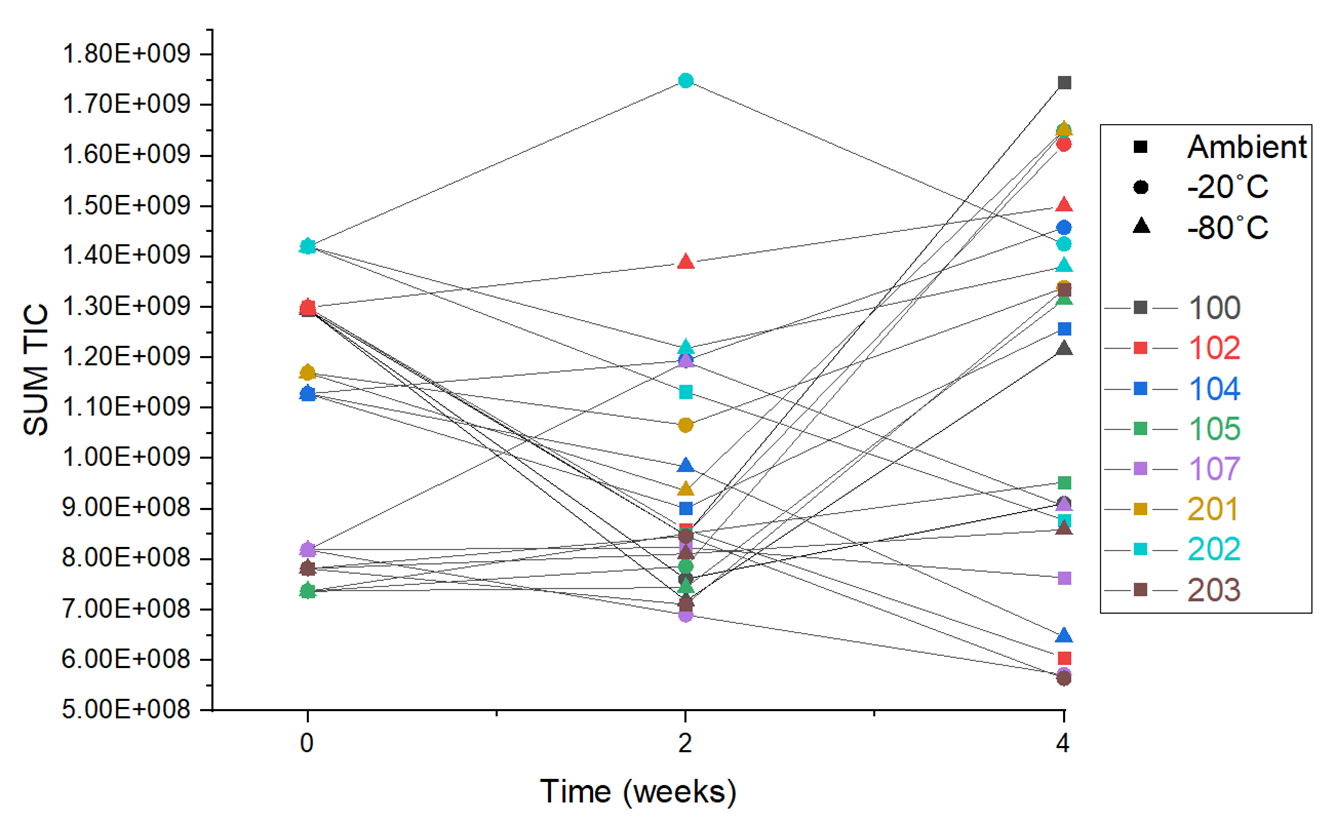


Figure SI 2: Sum of all peaks of TD-GC-MS chromatogram. In each case the t_0_ is fresh sebum and thus did not have a temperature of storage. The data shows no relation between total TIC and time or temperature.


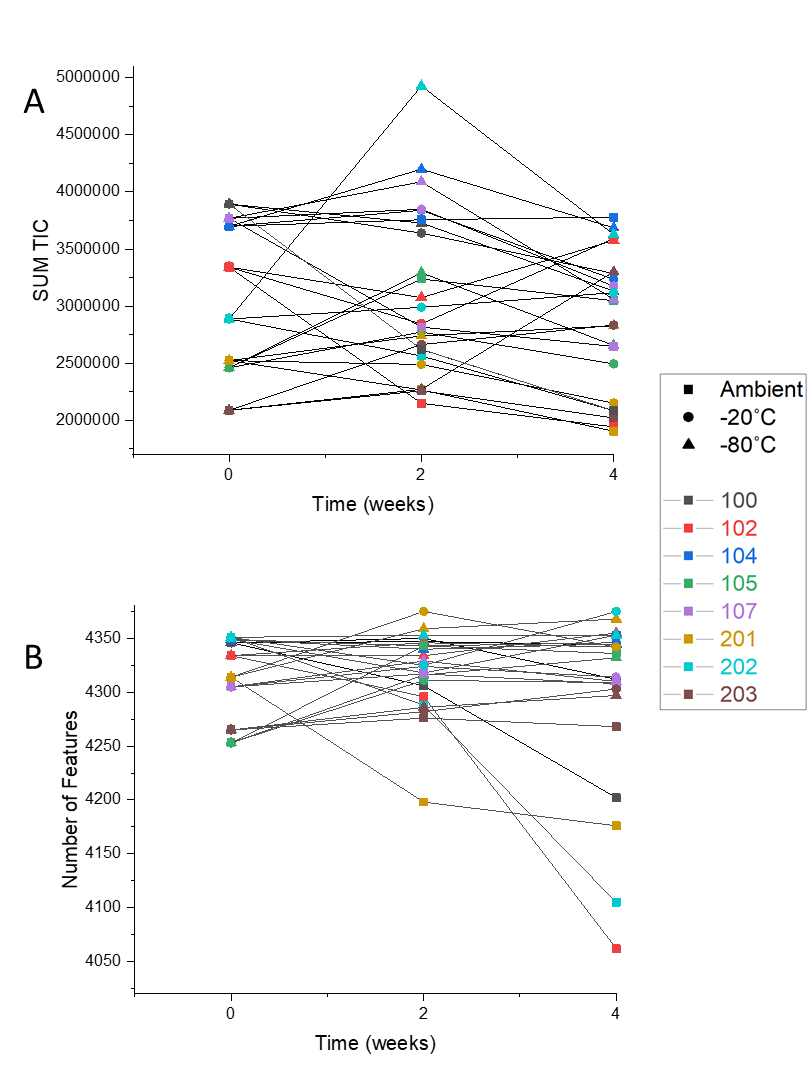


Figure SI 3: The total sum of all features (A) and total number of peaks (B) of the LC-IM-MS chromatograms. In each case the initial time point is ambient. There is no obvious trends in SUM TIC, though in number of features there is seen to be a decrease in half the participants over time at ambient temperatures.

Figure SI 4
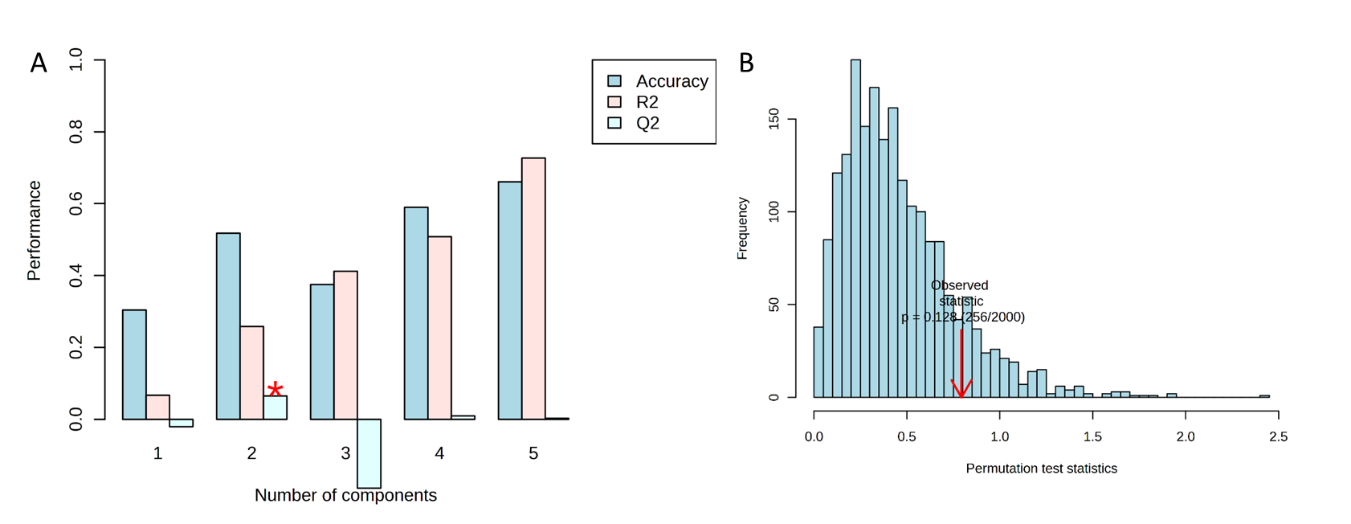
: PLS-DA for TD-GC-MS of sebum with respect to storage temperature was cross validated (A) using LOOCV and permutation testing (B) used n=2000.


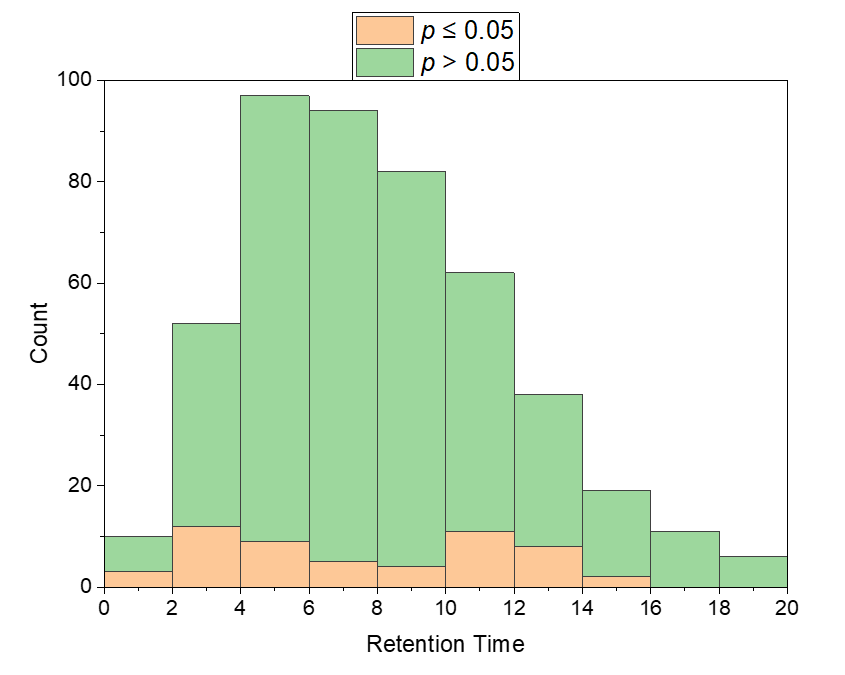
Figure SI 5: A histogram showing the features in the TD-GC-MS data plotted against retention time. Features found to be significant (p ≤ 0.05) with respect to storage temperature are shown in orange.


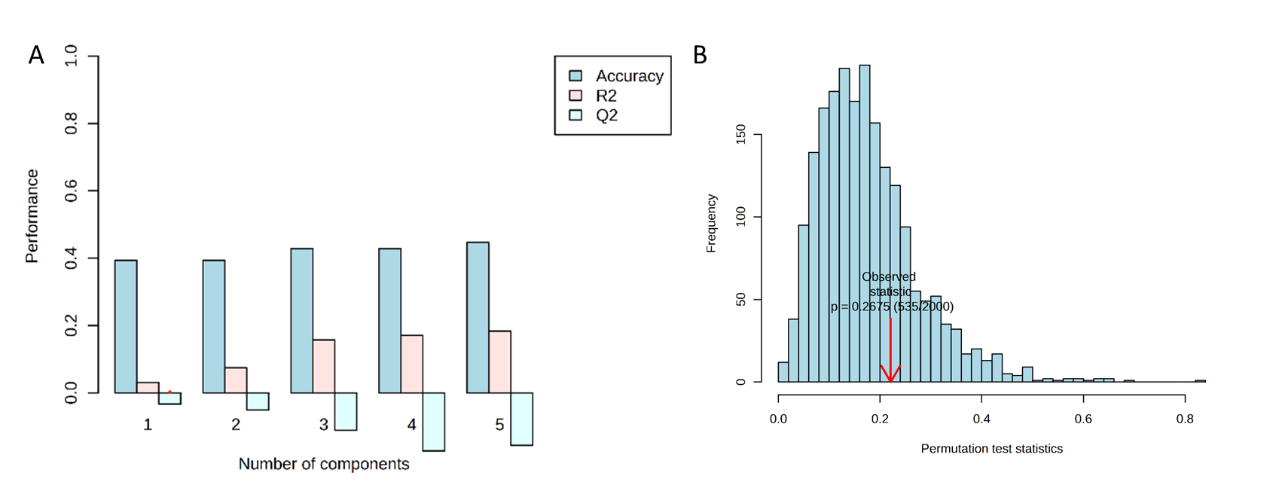


Figure SI 6: PLS-DA for LC-IM-MS of sebum with respect to storage temperature was cross validated (A) using LOOCV and permutation testing (B) used n=2000.

Table SI 1: Table of 20 features with lowest p values in LC-IM-MS as a function of storage temperature.

| Retention Time (mins) | *m/z* value | *p*-value | Regulation (with decreasing T) |
| --- | --- | --- | --- |
| 17.41 | 840.3778 | 5.17x10^-7^ | ↑ |
| 9.22 | 240.2323 | 7.08x10^-7^ | - |
| 16.08 | 785.7984 | 7.82x10^-7^ | ↑ |
| 8.58 | 212.2007 | 7.91x10^-7^ | ↓ |
| 6.44 | 219.1744 | 8.23x10^-7^ | - |
| 17.41 | 841.5404 | 8.55x10^-7^ | ↑ |
| 17.90 | 868.2129 | 1.26x10^-6^ | ↑ |
| 17.69 | 855.8822 | 1.97x10^-6^ | ↑ |
| 17.43 | 841.6127 | 2.01x10^-6^ | ↑ |
| 16.19 | 785.4925 | 2.34x10^-6^ | ↑ |
| 16.83 | 813.5912 | 2.37x10^-6^ | ↑ |
| 16.83 | 813.5129 | 3.20x10^-6^ | ↑ |
| 16.81 | 813.8327 | 3.72x10^-6^ | ↑ |
| 11.26 | 862.7245 | 3.80x10^-6^ | - |
| 16.83 | 812.2302 | 3.91x10^-6^ | ↑ |
| 15.97 | 795.9747 | 4.35x10^-6^ | ↑ |
| 15.61 | 779.1672 | 4.51x10^-6^ | ↑ |
| 17.67 | 854.1930 | 4.63x10^-6^ | ↑ |
| 9.88 | 230.2475 | 4.64x10^-6^ | ↓ |
| 13.32 | 616.5890 | 4.71x10^-6^ | - |


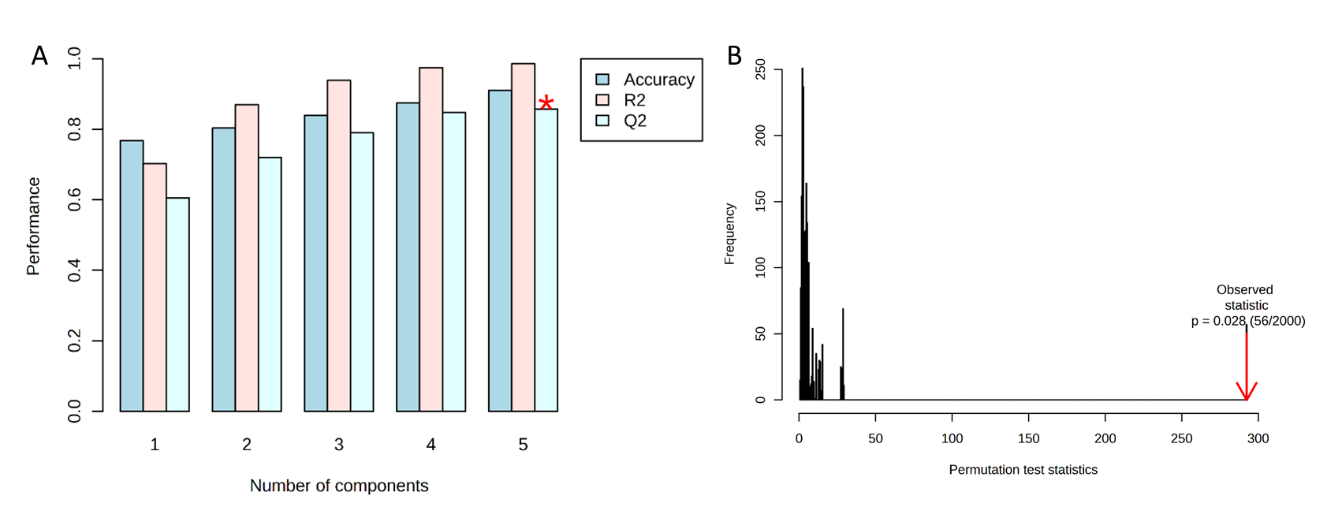


Figure SI 7: PLS-DA for TD-GC-MS of sebum with respect to storage time was cross validated (A) using LOOCV and permutation testing (B) used n=2000.

Table SI 2: For TD-GC-MS analysis with respect to storage time, the 20 features with the lowest p-value by ANOVA testing were putatively identified (MSI level 2) by the GOLM database. TMS derivatives and MF < 75 were not considered identified. A majority of these significant features increased over time and were identified as FAMEs.

| Retention Time (mins) | Putative ID | *p*-value | Regulation (with increasing time) |
| --- | --- | --- | --- |
| 2.33 | - | 9.35x10^-14^ | ↓ |
| 2.29 | Hexadecane | 2.13x10^-13^ | - |
| 2.17 | - | 5.84x10^-13^ | ↑ |
| 8.20 | Tridecanoic acid methyl ester | 1.10x10^-10^ | ↑ |
| 6.93 | Undecanoic acid methyl ester | 3.36x10^-10^ | ↑ |
| 7.86 | Dodecanoic acid methyl ester | 4.62x10^-10^ | ↑ |
| 7.53 | Dodecanoic acid methyl ester | 5.95x10^-10^ | ↑ |
| 8.97 | Tetradecanoic acid methyl ester | 3.10x10^-9^ | ↑ |
| 9.56 | Tetradecanoic acid methyl ester | 3.21x10^-9^ | ↑ |
| 7.94 | Tridecanoic acid methyl ester | 4.41x10^-9^ | ↑ |
| 11.19 | Tetradecanoic acid methyl ester | 6.93x10^-9^ | ↑ |
| 11.17 | Hexadecanoic acid methyl ester | 9.60x10^-9^ | ↑ |
| 9.48 | Pentadecanoic acid methyl ester | 9.80x10^-9^ | ↑ |
| 4.13 | Pentasiloxane | 1.17x10^-8^ | ↑ |
| 5.79 | Nonanoic acid methyl ester | 1.34x10^-8^ | ↑ |
| 9.36 | Tetradeanoic acid methyl ester | 1.63x10^-8^ | ↑ |
| 9.81 | Pentadecanoic acid methyl ester | 1.74x10^-8^ | ↑ |
| 10.38 | Hexaadecanoic acid methyl ester | 2.07x10^-8^ | ↑ |
| 8.81 | Tetadecenoic acid methyl ester | 6.40x10^-8^ | ↑ |
| 11.41 | Heptadecanoic acid methyl ester | 1.59x10^-7^ | ↑ |


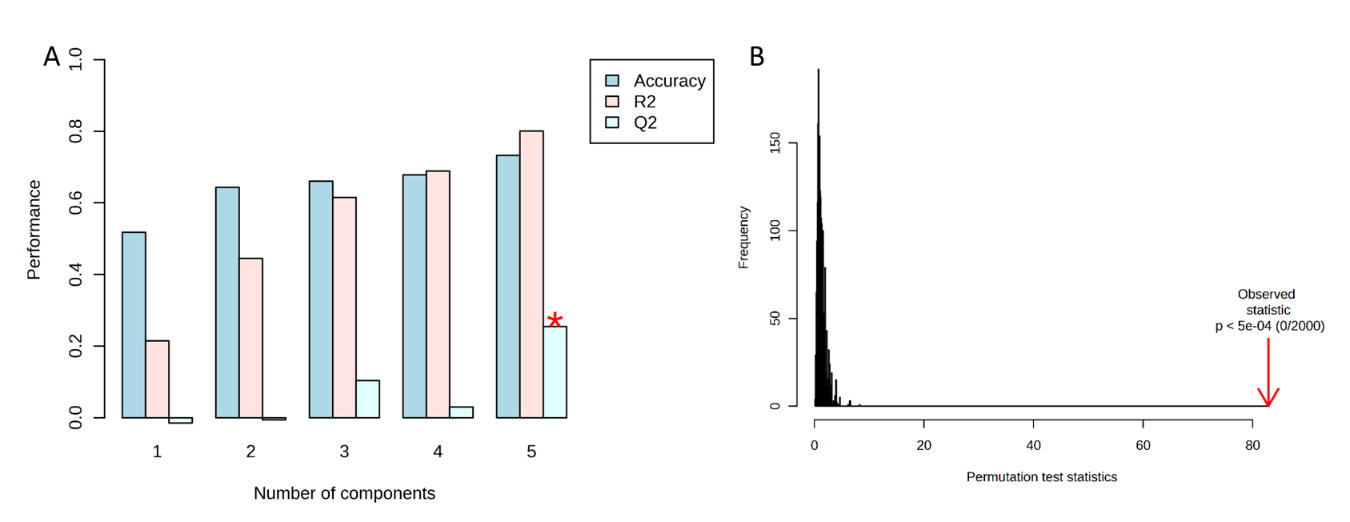


Figure SI 8: PLS-DA for LC-IM-MS of sebum with respect to storage time was cross validated (A) using LOOCV and permutation testing (B) used n=2000.

Table SI 3:Table of 20 features with lowest p values in LC-IM-MS as a function of time.

| Retention Time (mins) | *m/z* value | *p*-value | Regulation (with increasing time) |
| --- | --- | --- | --- |
| 9.35 | 1194.8195 | 1.13x10^-40^ | ↑ |
| 6.44 | 219.1744 | 7.53x10^-19^ | - |
| 2.47 | 546.8761 | 1.49x10^-18^ | ↓ |
| 2.49 | 986.6626 | 2.28x10^-15^ | ↓ |
| 2.49 | 502.8504 | 7.66x10^-15^ | ↓ |
| 2.49 | 478.4270 | 1.05x10^-14^ | - |
| 2.49 | 480.3292 | 1.70x10^-12^ | ↑ |
| 2.53 | 942.6363 | 1.95x10^-12^ | ↓ |
| 3.60 | 268.2633 | 3.12x10^-11^ | ↓ |
| 1.56 | 342.3721 | 3.34x10^-11^ | - |
| 2.53 | 458.8256 | 5.02x10^-11^ | ↓ |
| 2.47 | 1074.7138 | 8.46x10^-11^ | ↓ |
| 2.53 | 898.6100 | 1.41x10^-9^ | ↓ |
| 1.07 | 974.4565 | 3.07x10^-9^ | - |
| 11.97 | 401.4056 | 5.38x10^-9^ | - |
| 5.61 | 324.3255 | 6.38x10^-9^ | ↓ |
| 1.92 | 342.3724 | 7.83x10^-9^ | - |
| 9.69 | 486.4312 | 1.09x10^-8^ | - |
| 2.29 | 668.0747 | 1.82x10^-8^ | - |
| 1.83 | 340.3569 | 2.32x10^-8^ | - |


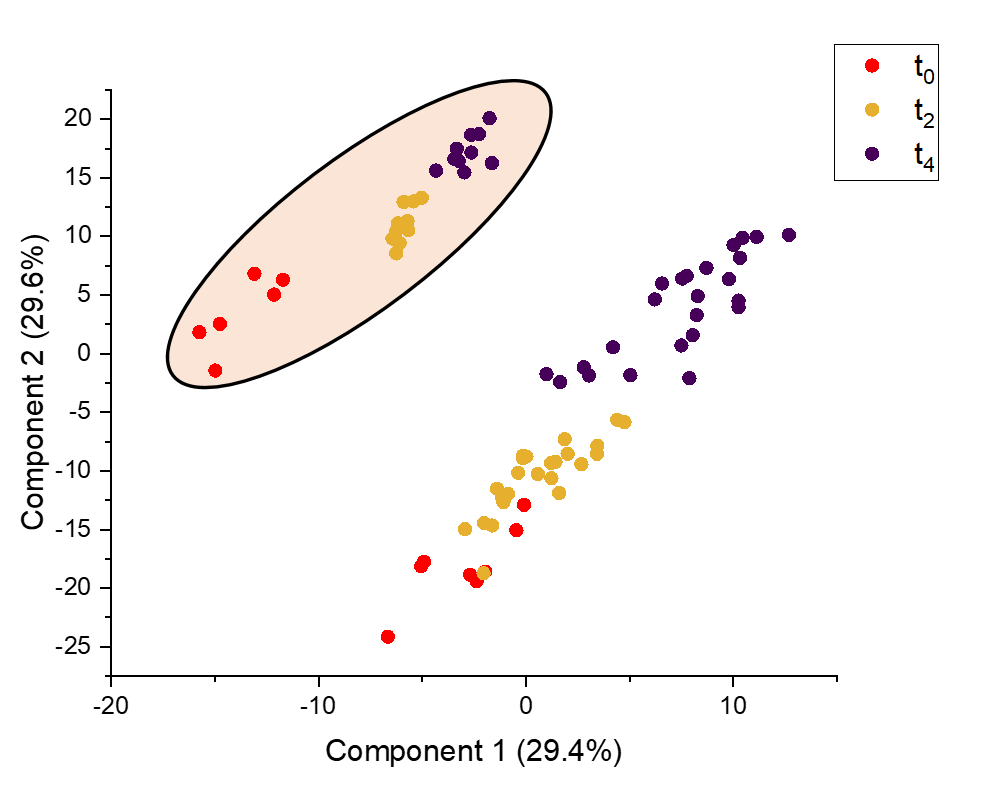


Figure SI 9: A PLS-DA scores plot of TD-GC-MS samples and SST injections over time points. Initial time t_0_ is represented in red, t_2_ in orange and t_4_ in purple. The points in the orange circle represent the SST. It can be seen in both SST and samples that there is clustering of the batches which can be attributed to instrument drift.

SI 6: Trend of Significant Features Across Participants

Due to the relatively small sample size, changes seen in one participant could bias results. To combat this, for each model we examined the four most significant features across the participants. We found the trend seen across the participants is consistent with the trend observed in the scores plots.

Storage Temperature

The top four features with the lowest *p*-value were selected for both the GC-MS and LC-MS analysis for further examination. In each case the samples stored at -20 °C were used.


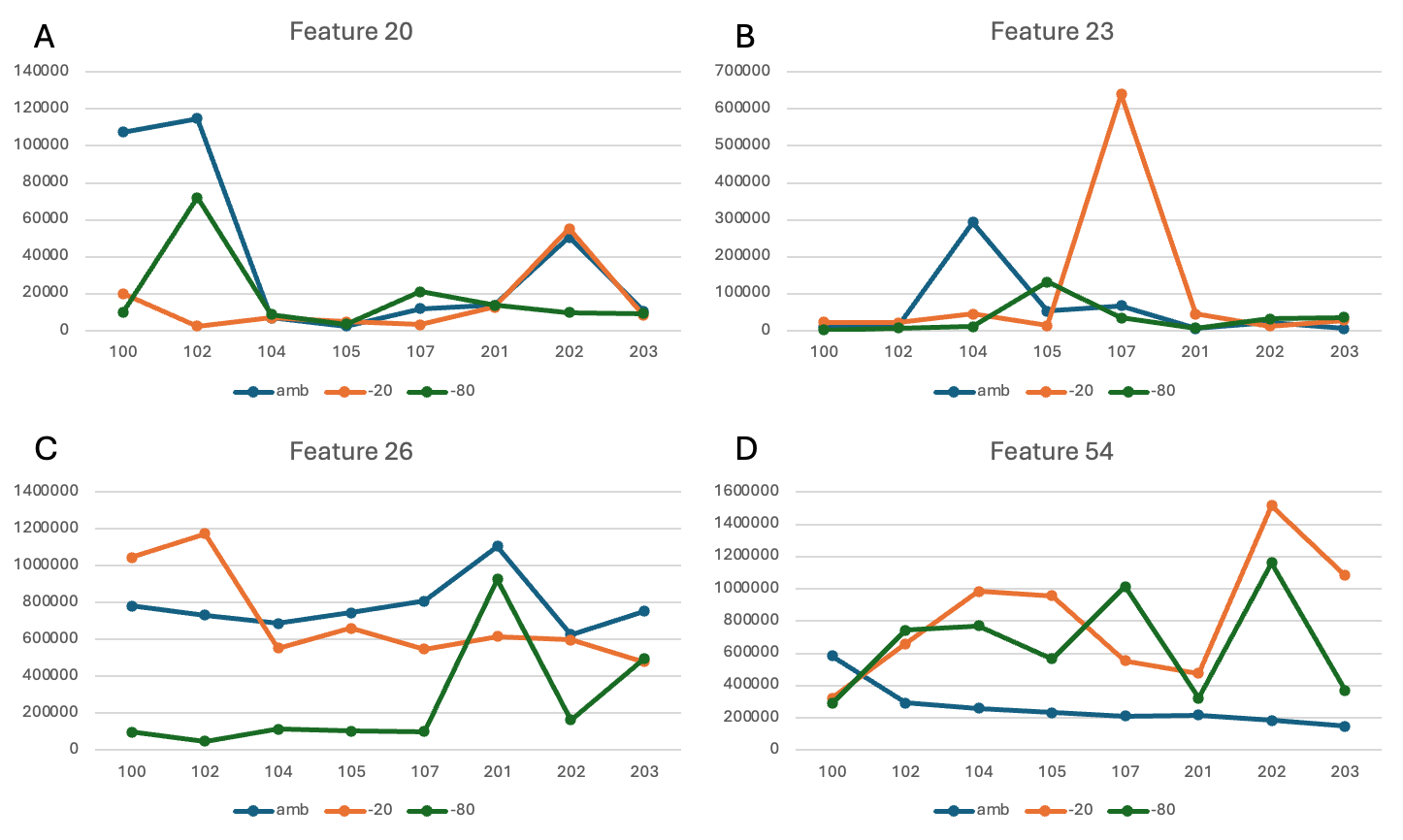


Figure SI 10: The four most significant features found in the TD-GC-MS matrix with respect to storage temperature are displayed by their intensity across the individual participants. It can be seen that none of the features show consistent pattern over any of the participants, which is in agreement with the supervised scores plots.

The features are shown as their intensity for each temperature condition for each individual. The four features both show there is no trend over the individual participants which is consistent with the supervised model and scores plots.


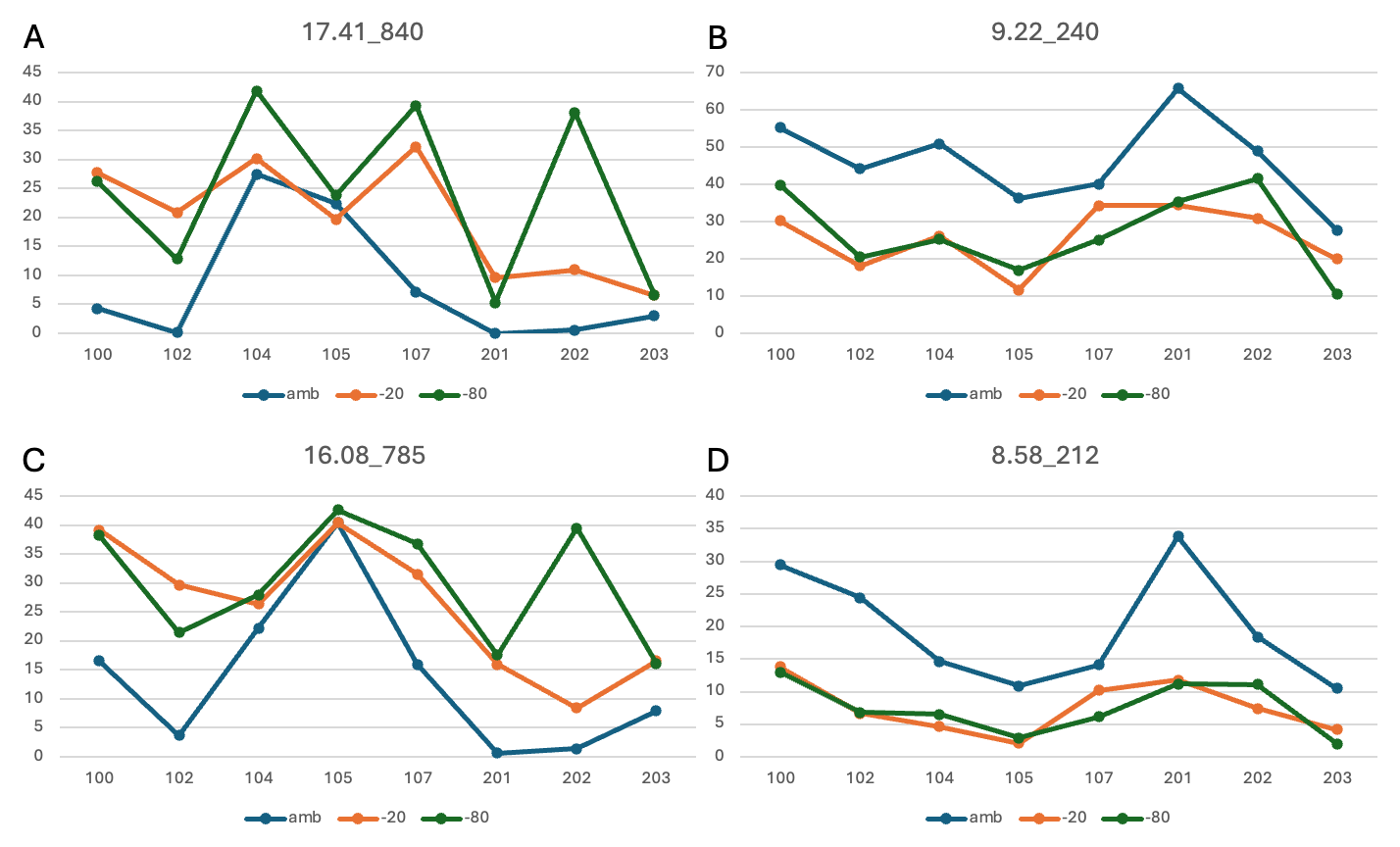


Figure SI 11: The four most significant features found in the LC-MS matrix with respect to storage temperature are displayed by their intensity across the individual participants. It can be seen that though none of the features show consistent pattern over any of the participants, the ambient point shows the most consistency in up and down regulation which is in agreement with the supervised scores plots.

The four selected features are shown as intensity across the participants. None of the features show consistency with all participants over the temperature range, though in B and D the intensities corresponding to ambient temperatures separate more from the other storage temperature conditions. This is consistent with the scores plots shown.

Length of Time of Storage

The top four features with the lowest *p*-value were selected for both the GC-MS and LC-MS analysis for further examination. In each case the samples stored at ambient conditions were selected.


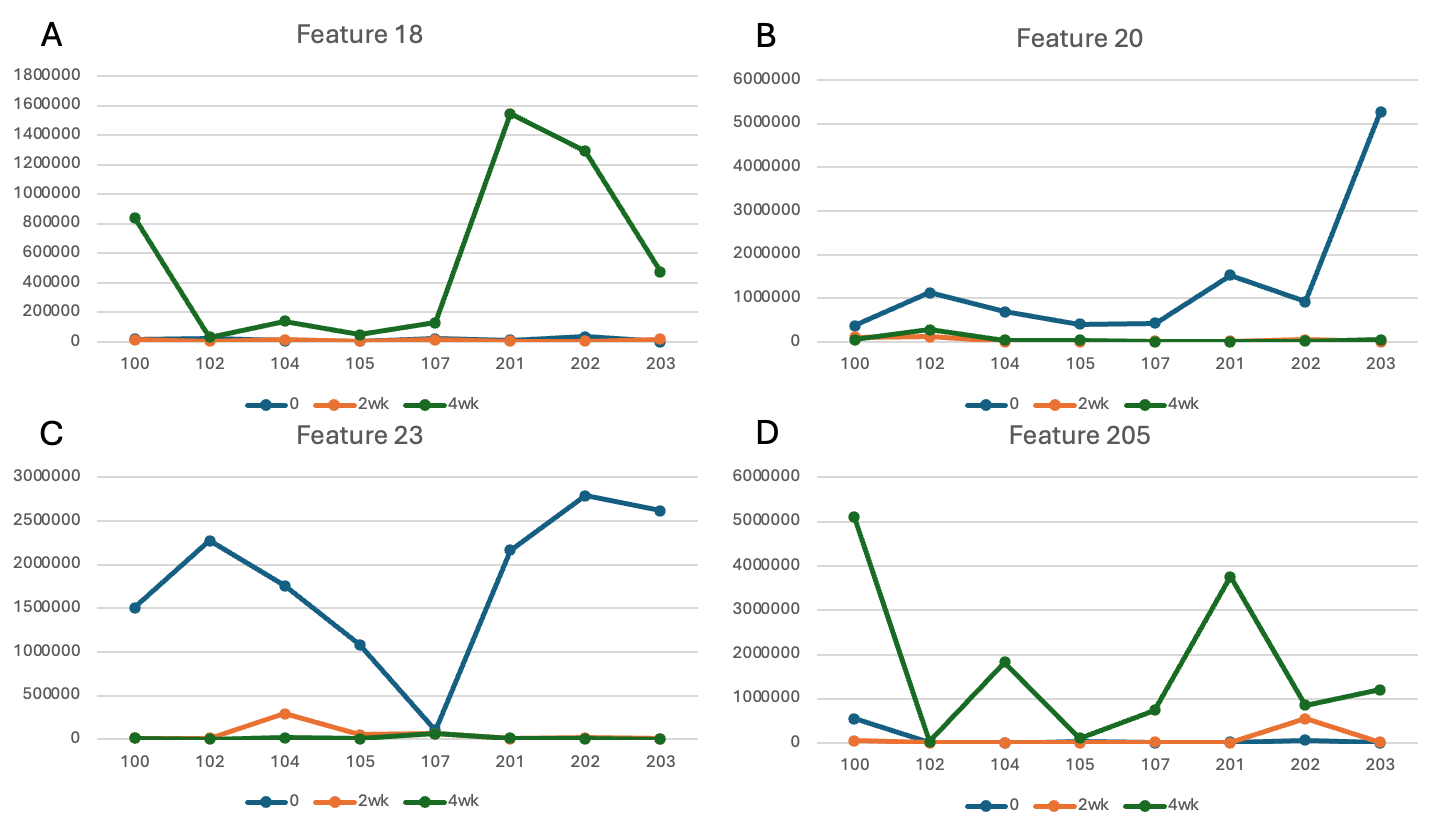


Figure SI 12: The four most significant features found in the TD-GC-MS matrix with respect to storage time are displayed by their intensity across the individual participants. It can be seen that some the features show a trend over the participants for instance in A and D the four week timepoint is upregulated and in B and C the timepoint zero is upregulated. This is consistent with the scores plots that show discrimination of the three timepoints.


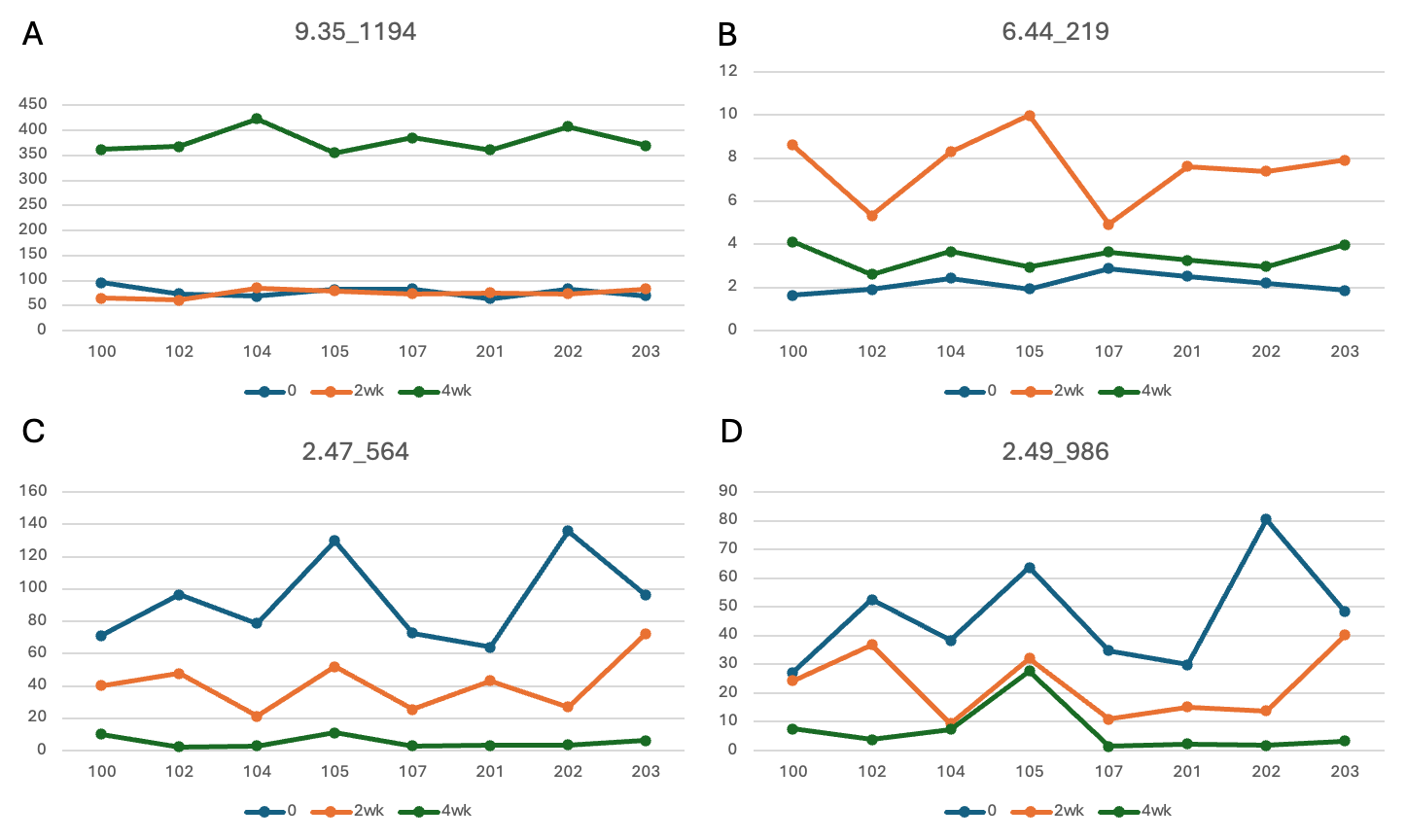


Figure SI 13: The four most significant features found in the LC-MS matrix with respect to storage time are displayed by their intensity across the individual participants. It can be seen that some the features show a trend over the participants for instance in A the four week timepoint is upregulated and in C and D the timepoint zero is upregulated. This is consistent with the scores plots that display clustering of the three timepoints.

The features found significant over the time points were extracted and plotted as intensity for each participant. Interestingly, the trends over the individuals seem to align well with the scores plots which showed the timepoints clustered for both TD-GC-MS and LC-MS.

References

(1) Waters Corporation. An Overview of the Principles of MSE, The Engine That Drives MS Performance. *Waters Appl. Note* **2011**, 1–6.

(2) Sinclair, E.; Walton-Doyle, C.; Sarkar, D.; Hollywood, K. A.; Milne, J.; Lim, S. H.; Kunath, T.; Rijs, A. M.; de Bie, R. M. A.; Silverdale, M.; Trivedi, D. K.; Barran, P. Validating Differential Volatilome Profiles in Parkinson’s Disease. *ACS Cent. Sci.* **2021**, *7* (2), 300–306. https://doi.org/10.1021/acscentsci.0c01028.
